# Concurrent histological lesions and molecular detection of porcine circovirus 3 in pigs with skeletal abnormalities and humpy-back posture

**DOI:** 10.1177/03009858251386914

**Published:** 2025-11-06

**Authors:** Giuliana Rosato, Grace M. Makoni, Àlex Cobos, Marina Sibila, Joaquim Segalés, Robert Graage, Dolf Kümmerlen, Thomas Echtermann, Nadja Aeberhard, Hanna Marti, Barbara Helminger, Frauke Seehusen

**Affiliations:** 1 University of Zurich, Zurich, Switzerland; 2 Universitat Autònoma de Barcelona (UAB), Bellaterra, Spain; 3 WOAH Collaborating Centre for the Research and Control of Emerging and Re-Emerging Swine Diseases in Europe (IRTA-CReSA), Bellaterra, Spain; 4 Qualiporc Health Service, Qualiporc Cooperative, Appenzell, Switzerland

**Keywords:** humpy-back syndrome, in situ hybridization, porcine circovirus 3, quantitative real-time PCR

## Abstract

Porcine circovirus 3 (PCV-3) is associated with various pathological conditions, including systemic disease and reproductive disorders; however, its role in skeletal abnormalities has never been elucidated. This study included 36 cases displaying spinal malformations, rib swelling, head edema, gait abnormalities, and/or increased late-term abortions. Investigated animals consisted of 9 aborted fetuses, 9 piglets, 12 weaners, and 6 finishers. Histologically, PCV-3 associated lesions were identified in 23/36 cases (64%), including (peri-)arteritis and rib fractures with prominent callus formation. Central nervous system (CNS) lesions, in addition to vascular changes, comprised meningoencephalitis and gliosis. Thirteen animals (36%) did not display histological lesions. PCV-3 DNA was detected by real-time PCR (qPCR) in 25/36 animals (69%), with high viral loads in the bone and CNS. Three aborted fetuses tested positive for PCV-3 despite lacking macroscopic and histologic lesions. *In situ* hybridization (ISH) revealed the presence of PCV-3 RNA in multiple organs, including arteries, the heart, CNS, and bone. Signals were detected in periosteal arteries and osteoblasts, within calluses, and in arteries within the surrounding skeletal muscles. This study strengthens the association between PCV-3 and multisystemic inflammatory diseases, expanding its known pathogenicity to include skeletal lesions and spinal deformities. It is the first documentation of PCV-3 genome in histologically altered bone. This finding could suggest a possible etiological role in musculoskeletal abnormalities. In addition, this study is the first to report PCV-3-associated lesions in slaughter-ready finisher pigs. The integration of histological investigations, PCR, and ISH techniques is essential for the diagnosis of PCV-3-associated diseases and related lesions.

Circoviruses are small, non-enveloped viruses with single-stranded, circular DNA genomes that are known to infect a broad range of animals. Currently, 4 distinct porcine circovirus species (PCVs) have been identified in swine, classified numerically based on the order of their discovery: PCV-1, PCV-2, PCV-3, and PCV-4. Porcine circovirus 3 (PCV-3) was first identified in the United States in 2015 through metagenomic analysis of sows exhibiting lesions resembling porcine dermatitis and nephropathy syndrome, along with reproductive disorders. The viral genome was also detected in piglets showing myocarditis and systemic vascular inflammation.^37,42^ Since its discovery, the virus has been identified globally^2,4,6,17,21,22,29,32,43,52,61^ in both healthy pigs and those exhibiting various clinical signs, including reproductive disorders, porcine dermatitis and nephropathy syndrome,^37^ multisystemic inflammatory disease,^42^ respiratory disorders,^26,59^ enteric disorders,^59,60^ and neurological signs.^3,7^

Histological lesions observed in naturally occurring PCV-3 infections include myocarditis, nephritis, and (peri-)arteritis affecting the cardiac, renal, and mesenteric arteries.^3,5,10,35,37,42,44^ In addition, experimental infection studies have reported arteritis in the same tissues,^5,9,35,54^ although this finding was not consistent across all of them.^13^

Detection of PCV-3 in histologic lesions in diseased pigs has led to recommendations for establishing case definitions and diagnostic criteria for 2 distinct PCV-3 associated diseases.^46^ The first, referred to as PCV-3 reproductive disease (PCV-3-RD), is mainly characterized by increased stillbirths, mummified fetuses, and weak piglets that may die within few days after birth.^3,34,37,49^ The second, termed PCV-3 systemic disease (PCV-3-SD), occurs in postnatal pigs and is associated with growth retardation, wasting, and anorexia.^3,10,42^ The diagnostic criteria for both (PCV-3-RD and PCV-3-SD) include compatible clinical signs, characteristic histologic lesions (e.g. multisystemic [peri-]arteritis), and the presence of moderate to high viral loads in the lesions. A recent experimental study^9^ suggests that PCV-3-SD likely reflects a persistent infection in piglets exposed to PCV-3 *in utero*. Cases of natural infection have shown that piglets born to litters with PCV-3-RD can reach weaning age and display signs of systemic disease,^33^ supporting the notion that PCV-3-RD and PCV-3-SD may not be different conditions.

Studies have implicated PCV-3 in reproductive failure in swine, particularly in cases of fetal mummification and stillbirth. While a definitive causal relationship has not yet been established, multiple reports provide substantial evidence linking the presence of PCV-3 with lesions and high viral loads in fetuses from affected sows,^3,5,35,48,49^ as well as indications of vertical transmission.^56^

The association of PCV-3 with neurological signs and corresponding histological lesions in the CNS has been documented in multiple studies.^3,7,35,42^ Among the neurological signs, congenital tremor has been observed,^3,7,35^ although some of these studies reported co-infections with other pathogens. Histological lesions described include meningitis,^42^ gliosis, and perivascular lymphocytic cuffing.^3,35^ Experimental infection with PCV-3 has induced CNS lesions consisting of lymphoplasmacytic encephalitis^54^ with glial foci and perivascular cuffing in five-week-old piglets and in the offspring of infected pregnant gilts,^9^ respectively. The presence of PCV-3 viral genome within CNS lesions was confirmed by the authors.^9,54^ However, these CNS lesions were consistently observed alongside other lesions characteristic of PCV-3-SD.

Only a limited number of published studies have demonstrated the presence of PCV-3 within tissue lesions using *in situ* hybridization (ISH), suggesting a potential association between the virus and the etiology of these lesions.^1,3,10,12,27,33,34,42,44,49,56^

Kyphosis and lordosis are pathological conditions that manifest as abnormal curvature of the spinal column. Kyphosis is typically absent at birth but generally becomes visible in pigs between 8 and 16 weeks of age^53^ and often results from underlying primary lesions in the vertebrae, such as osteomyelitis, fractures, neoplasms, and metabolic diseases.^31^ While the condition is frequently classified as idiopathic, evidence suggests potential contributions from congenital, hereditary, or nutritional factors.^23^ Other proposed predisposing factors include painful musculoskeletal conditions affecting the limbs and back,^30^ musculo-mechanical stress on the lumbar spine,^11^ or early-onset puberty in male pigs.^14^ Although the condition occurs sporadically within herds, primarily affecting individual animals, outbreaks have been reported, with incidence rates up to 30%–50%.^11,15,38,40,55^ The curvature is most frequently observed at the level of the 14th–16th thoracic vertebrae and has been associated with abnormal epiphyseal ossification.^36^ Genetic studies indicate a moderate hereditability for this condition, with multiple loci implicated in its development.^25^

The early onset of “humpy-back” in pigs younger than the typical ages for spinal deformities was first documented by Penny et al^41^ on farms in the UK. Clark^8^ later provided a detailed description of the syndrome in piglets aged 1–12 weeks from Canadian farms. Affected animals exhibited signs of depression, displayed both lordosis and kyphosis, and, in some cases, had caudally rotated ears. Rib fractures and prominent bone calluses were frequently observed. Histological examination revealed lymphocytic and/or necrotizing (peri-)arteritis in multiple organs, along with lymphocytic myositis and myocarditis.^8^ Similar cases of piglets displaying a “humpy-back” posture, rib fractures, and histopathological findings of (peri-)arteritis have been documented by other researchers.^16,38^ One study additionally reported the presence of alopecia areata in affected animals.^16^

In March 2023, our laboratory was contacted to investigate an outbreak on a Swiss breeding farm (referred to as farm A in the following text), where 4- to 5-week-old piglets exhibited spinal deformities, specifically an upward curvature of the lumbar spine (kyphosis) and a downward curvature of the thoracic spine (lordosis), commonly known as “humpy-back posture.” In addition, the herd veterinarian reported that the piglets and weaner pigs on the farm had thickened ribs, caudally rotated ears colloquially referred to as “Dumbo ears” or “flying pigs,” and facial edema, despite otherwise unremarkable clinical condition. Histological examination of the submitted piglets revealed lesions compatible with PCV-3-SD.

The aim of the present study was to investigate the occurrence of PCV-3 infection using quantitative PCR (qPCR) and ISH in samples from pigs with spinal deformities (“humpy-back” posture), caudally rotated ears, swollen ribs, and CNS signs. In addition, samples from all animals originating from farm A, including aborted fetuses, were analyzed regardless of the symptoms. Furthermore, we aimed to determine whether there is an association between the observed musculoskeletal lesions and PCV-3 infection.

## Material and Methods

This study, carried out between March 2023 and August 2024, included 36 pigs that were selected based on their clinical signs or their farm of origin. The clinical presentations investigated comprised “humpy-back” posture, swollen ribs, head edema, neurological signs, and abortions occurred during the study period in farm A.

A total of 20 pigs underwent full necropsy at the Institute of Veterinary Pathology, Vetsuisse Faculty, University of Zurich. In addition, 4 animals from farm A were fattened, slaughtered in-house, and subsequently sampled. For the remaining 12 pigs, herd veterinarians performed necropsies and sampling on the farm. In these cases, only formalin-fixed tissues were available. Tissues sampled for further analysis included bones (vertebrae and ribs), brain and spinal cord, kidney, mesenteric lymph nodes and associated vascular plexus, and heart. Formalin-fixed tissues were utilized for histological evaluation, while additional tissue samples were stored at −20°C for DNA extraction and subsequent qPCR testing.

Hematoxylin and eosin–stained tissues were histologically evaluated in all cases, with a focus on lesions known to be caused by PCV-3 infection, such as (peri-)arteritis. All cases were tested for the presence of PCV-3 by qPCR, following previously described protocols.^44^ Target tissues included kidney, mesenteric lymph nodes, and heart. Bone and CNS tissue were also analyzed if available. Selected formalin-fixed tissues were incorporated into tissue microarrays to facilitate high-throughput histological and molecular analyses. This method has been validated in prior studies.^20,44^ RNA-ISH for PCV-3 (*Circoviridae* SFpork/USA/2010 isolate; SFpork8 Rep protein-like gene complete sequence; PCV-3 viral nucleic acid [2–1049 region of ORF1 gene, GenBank: HQ839721.1]) was performed using RNAscope on 21 cases, selected based on the occurrence of histopathological lesions or either low or high viral load within tissue sections, as previously described.^44^

## Results

### Animal Data and Clinical Signs

Of the 36 submitted cases, 26 animals originated from farm A, which reported clinical signs including spinal column malformations, swollen ribs, head edema, gait abnormalities, and an increased rate of abortions during the last third of gestation. The remaining 10 unrelated cases were from 7 distinct Swiss farms reporting signs such as abnormal spinal column curvature, congenital tremors, head swelling, and/or caudally rotated ears. Of the 26 cases from farm A, 9 cases involved aborted fetuses at 3 different time points and from 3 different gilts, 2 involved suckling piglets, 11 involved weaning piglets, and 4 involved finishing pigs that were fattened and subsequently slaughtered on-site. Among the 10 unrelated cases from other farms, 7 involved suckling piglets, 1 involved a weaning piglet, and 2 involved finishers.

### Macroscopic and Histopathological Findings

**Figure 1.**
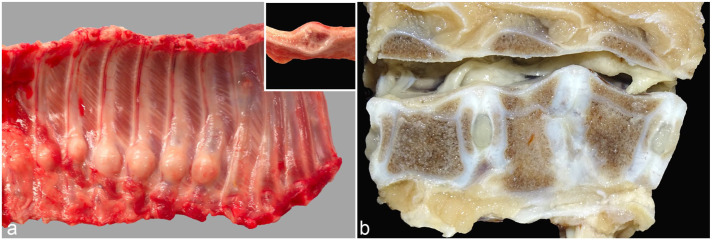
Macroscopic osseous lesions of a 5-week-old weaned piglet. (a) Left thoracic rib cage showing 7 adjacent ribs with prominent callus formation. Inset: longitudinal section through a callus. (b) Absence of the intervertebral disk between L1 and L2, with direct contact between the caudal epiphyseal cartilage of L1 and the cranial epiphyseal cartilage of L2. L1 exhibits an irregular vertebral body with a widened ventral contour, while L2 displays a ventrally shortened, wedge-shaped vertebra. Mild narrowing of the spinal canal is evident due to dorsal cartilaginous proliferation.

Macroscopic examination of the animals (*n* = 17) with a documented history of clinical signs, including rib swelling and abnormal spinal curvature (Supplemental Fig. S1), revealed multiple rib fractures of varying severity, accompanied by pronounced callus formation (Fig. 1a). The fractures were randomly distributed, occurred unilaterally or bilaterally, involved various costal levels, were not associated with the costochondral junctions, and affected between 1 and 8 ribs per animal. In addition, 2 finishing pigs exhibited macroscopic lesions affecting the vertebral bodies in the lumbar spine region (Fig. 1b). The lesions were characterized by irregular vertebral architecture, absence of the intervertebral disk, direct contact between adjacent epiphyseal cartilage plates, and mild narrowing of the spinal canal due to dorsally oriented cartilaginous proliferation. No macroscopic lesions were observed in any of the 9 aborted fetuses.

**Figure 2.**
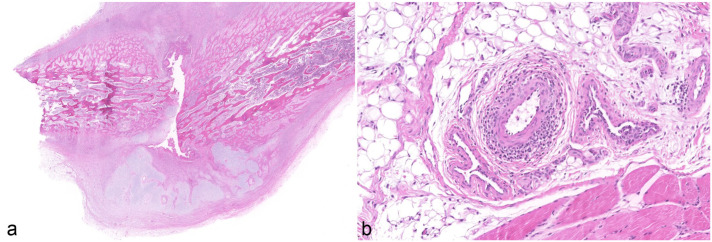
Histopathological osseous lesions of the same 5-week-old weaned piglet as shown in Figure 1. Hematoxylin and eosin. (a) Rib fracture with irregular callus formation, formation of woven bone and chondroid tissue, and severe remodeling. (b) Rib. Moderate lymphohistiocytic and plasmacytic (peri-)arteritis in surrounding adipose tissue and skeletal muscle.

Of the 36 cases, 23 (64%) exhibited histological lesions consistent with PCV-3-SD in one or more organs. These cases predominantly manifested as moderate to severe perivascular lymphohistiocytic to plasmacytic infiltrates within the tunica media and tunica adventitia of small- and medium-caliber arteries, particularly within the vascular mesenteric plexus, kidneys, and heart. In addition, in cases where available, histological examination of macroscopically altered ribs revealed chronic rib fractures accompanied by pronounced, irregular callus formation (Fig. 2a). Associated with these osseous lesions, the costal periosteal arteries and the arteries within the adjacent skeletal muscle also exhibited lymphohistiocytic to plasmacytic (peri-)arteritis (Fig. 2b). Histological examination of the skeletal system also included rib sections without macroscopically visible callus formation. In these areas, no histological evidence of metabolic bone diseases such as rickets or fibrous osteodystrophy was observed. Physiological growth plate morphology was preserved, and bone mineralization appeared normal (Supplemental Fig. S2). In the CNS, non-suppurative meningoencephalitis, multifocal gliosis, and perivascular cuffing were noted.

Thirteen animals (36%), including all 9 aborted fetuses and 4 suckling piglets, did not exhibit any histological lesions. Except for the identified inflammatory vascular lesions of (peri-)arteritis in multiple organs, no further histopathological alterations compatible with PCV-3 infection, such as myocarditis, as reported in the literature,^10,12,34^ were observed in the examined cases.

### qPCR Results

All 36 cases underwent qPCR analysis of mesenteric lymph nodes, kidney, and heart. In addition, when available and based on macroscopic and histological findings, CNS (brain and spinal cord) and bone were tested using either frozen tissues or formalin-fixed paraffin-embedded samples. Overall, 25 animals (69%) tested positive for PCV-3 DNA in 1 or more organs, with viral loads ranging from 8 copies/100 ng DNA to over 11 million copies/100 ng DNA. Bone tissue analysis revealed positive results in 9 of the 12 animals (75%) tested, with viral loads ranging from 14 copies/100 ng DNA to over 790,000 copies/100 ng DNA (Table 1). qPCR analysis of the CNS identified PCV-3 DNA in 10 of the 12 animals (83%) tested, with viral loads ranging from 1,435 copies/100 ng DNA to over 183,000 copies/100 ng DNA (Table 2).

**Table 1.** Quantitative PCR (qPCR) results (viral loads) in cases with skeletal lesions, including farms of origin.

Case	Farm of Origin^a^	Age (w)	PCV-3 Viral Load (Copies/100 ng DNA)
1	0	5	134
2	0	4	18,971
3	0	5	101
13	1	17	neg
14	0	30	neg
20	0	7	791,155
21	0	6	127
22	1	4	9,059
23	1	9	14
27	0	6	14
29	0	6	neg
30	0	1	271,879

Abbreviations: w, week; PCV-3, porcine circovirus 3; neg, no detection of PCV-3 by qPCR.

a 0 = Farm A; 1 = Other Swiss farm.

**Table 2.** Quantitative (qPCR) results (viral loads) in cases with central nervous system symptoms and lesions, including farms of origin.

Case	Farm of Origin^a^	Age (w)	PCV-3 Viral Load (Copies/100 ng DNA)
1	0	5	146,081
2	0	4	102,496
3	0	5	60,945
14	0	30	7,452
15	0	30	183,576
19	0	5	neg
20	0	7	2,319
21	0	6	neg
22	1	4	3,753
23	1	9	7,562
27	0	6	49,081
28	0	6	1,435

Abbreviations: w, week; PCV-3, porcine circovirus 3; neg, no detection of PCV-3 by qPCR.

a 0 = Farm A; 1 = Other Swiss farm.

Despite the absence of histological lesions, 3 (33%) of the 9 submitted aborted fetuses from 2 different time points and 2 different gilts tested positive for PCV-3, with viral loads ranging from 8 copies/100 ng DNA to 16,943 copies/100 ng DNA. Two animals tested positive only in the mesenteric lymph nodes, while 1 animal exhibited viral DNA presence in the mesenteric lymph nodes, heart, and kidney.

### ISH Results

A subset of 19 qPCR PCV-3-positive animals, along with a negative control group of 3 animals without viral DNA detection by qPCR (representing cases of abortions, CNS signs, and skeletal lesions), were selected for ISH analysis to determine the presence of PCV-3. These cases were chosen based on clinical signs, farm of origin, the organs exhibiting histological lesions, lesion types, and the age of the animal. In total, ISH was performed in 21 cases, covering 46 organs. All 3 qPCR-negative cases (6 organs) were also negative by ISH. Among the 18 qPCR-positive cases, 40 organs were examined by ISH. Of these, 13 cases (72%) comprising 33 qPCR-positive organs showed a high concordance, with 31 organs (78%) positive by ISH and two organs from two different animals (1 brain and 1 kidney) that were qPCR-positive but ISH-negative. In contrast, 5 cases (7 qPCR-positive organs) were entirely negative by ISH, although in one of these cases, a bone sample was also negative by qPCR. The qPCR-positive organs that did not show ISH labeling had viral loads ranging from 8 to 7,591 copies/100 ng DNA.

**Figure 3.**
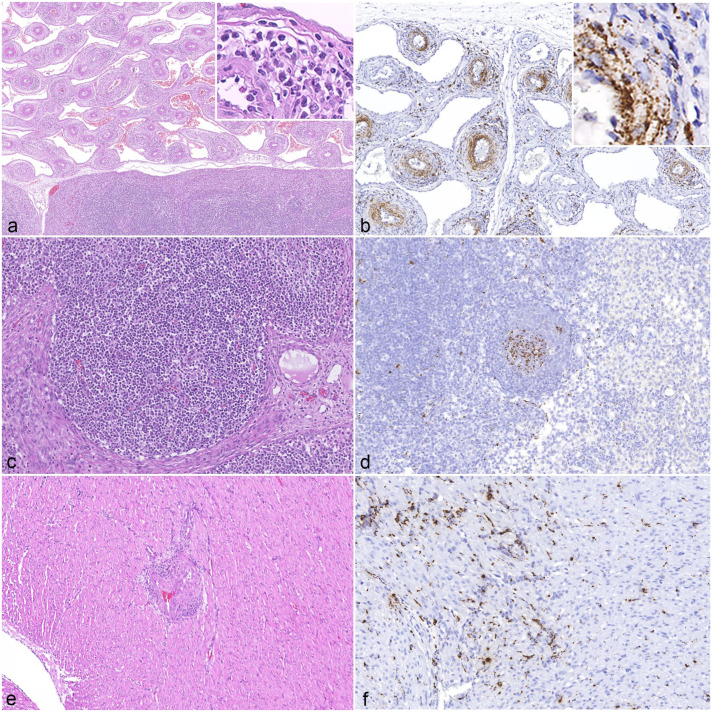
Histologic lesions of porcine circovirus-3 (PCV-3)-systemic disease. (a) Vascular mesenteric plexus of a 5-week-old weaned piglet. Severe lymphohistiocytic to plasmacytic (peri-)arteritis. Inset: higher magnification of the inflammatory infiltrate. Hematoxylin and eosin (HE). (b) Vascular mesenteric plexus of the same animal as in (a). Marked PCV-3 *in situ* hybridization (ISH)-positive signal in smooth muscle-like cells, macrophages, and possibly other mesenchymal cell types. Inset: higher magnification of PCV-3-positive cells in arterial wall. PCV-3 ISH. (c) Mesenteric lymph node of a 7-week-old weaned piglet. Lymphoid follicle without lesions. HE. (d) Mesenteric lymph node of the same animal as in (c). Moderate PCV-3-positive signal in germinal center. PCV-3 ISH. (e) Heart, myocardium of a 4-day-old suckling piglet. Moderate lymphohistiocytic to plasmacytic (peri-)arteritis. HE. (f) Heart of the same animal as in (e). Moderate PCV-3-positive signal in cardiomyocytes. PCV-3 ISH.

**Figure 4.**
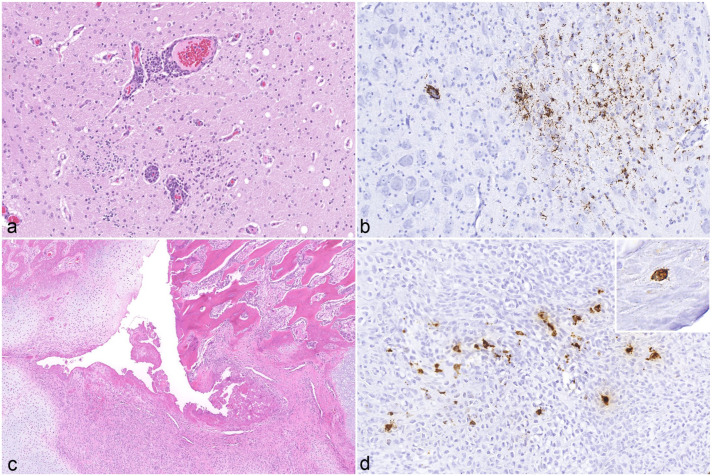
Histologic lesions in the brain and bone of a 5-week-old weaned piglet. (a) Cerebrum. Multifocal gliosis and lymphohistiocytic perivascular cuffing. Hematoxylin and eosin (HE). (b) Cerebrum. Moderate porcine circovirus-3 (PCV-3)-positive signal in microglia and neurons. PCV-3 *in situ* hybridization (ISH). (c) Rib, fracture. Disorganized callus formation characterized by fibrovascular tissue interspersed with fibroblasts and chondroblasts. HE. (d) Rib. Moderate PCV-3-positive signal in fibroblasts within the callus and in osteoblasts (inset). PCV-3 ISH.

PCV-3 ISH signals were detected in association with vascular inflammatory lesions. Arteries with consistent histological lesions (Fig. 3a) demonstrated segmental or circumferential PCV-3 labeling, which appeared to be localized in smooth muscle-like cells, macrophages, and possibly other mesenchymal cells (Fig. 3b). In lymph nodes, ISH signals were observed in the germinal centers of lymph follicles (Fig. 3d). In the heart, widespread PCV-3 labeling was noted in cardiomyocytes (Fig. 3f). In the CNS, ISH signal was identified in glial cells and, occasionally, in neurons (Fig. 4b). In bone tissue, which was examined in 4 cases (3 qPCR-positive and 1 qPCR-negative), ISH-positive signals were detected in all qPCR-positive bones, while the qPCR-negative bone was also negative by ISH. Signals were detected in the walls of the periosteal arteries, scattered single cells within the medullary cavity, osteoblasts, and fibroblasts surrounding the fracture callus (Fig. 4d).

Not all tested tissues, however, exhibited positive ISH signals. A summary and comparative analysis of the qPCR and ISH results are presented in Table 3. All animal data are presented in Supplemental Table S1.

**Table 3.** Summary of quantitative PCR (qPCR) and *in situ* hybridization (ISH) results for different tissues categorized by age groups.

Age Group	N (Total Cases)	PCV-3 qPCR Positive Cases/N (% Positive)	PCV-3 ISH Positive Cases (Positive Organs/N Analyzed)	Tissues With Positive ISH Signals
Aborted Fetuses	9	3/9 (33%)	LN: 0/2	None
Piglets	8	4/8 (50%)	LN: 3/3	Vascular plexus, germinal centers
			K: 4/4	Interstitial arteries
Weaners	13	12/13 (92%)	LN: 6/8	Vascular plexus, germinal centers
			K: 3/4	Interstitial arteries
			H: 2/3	Cardiomyocytes
			Bo: 3/3	Osteoblasts, medullary cavity, periosteal arteries, skeletal muscle arteries
			CNS 4/6	Microglia, neurons, meningeal and parenchymatous arteries
Finishing Pigs	6	6/6 (100%)	LN: 1/1	Vascular plexus
			K: 2/3	Interstitial arteries
			Bo: 0/1	
			CNS: 0/1	

Abbreviations: PCV-3, porcine circovirus 3; LN, lymph node; K, kidney; H, heart; Bo, bone; CNS, brain or spinal cord.

## Discussion

This study highlights previously undocumented aspects of PCV-3 infections, with histological lesions observed aligning with established descriptions of PCV-3-SD. A novel finding is the detection of the virus within bone lesions, specifically chronic rib fractures accompanied by pronounced, irregular callus formation, which has not been reported in previous studies on PCV-3 infection.

Over the past two decades, independent research from different countries has documented cases of pigs exhibiting a “humpy-back” posture, accompanied by macroscopic features such as caudally rotated ears, multiple chronic rib fractures,^8^ and histologically identifiable inflammatory vascular lesions.^8,16,38^ These findings are strikingly similar to those observed in our study. At the time of these publications, PCV-3 had not yet been discovered or isolated. Therefore, the authors hypothesized that these earlier cases, which exhibited similar clinical signs and histopathological lesions, might have been associated with a PCV-3 infection. Given the virus’s ancient origin, as indicated by the literature,^20^ it may be possible that PCV-3 could have been present in these cases before its discovery but has not been systematically studied in this context.

To the authors’ knowledge, detection of PCV-3 by ISH has never been reported in osseous tissues prior to this study. This finding is significant, as it may represent a possible novel aspect of PCV-3 pathogenesis. While previous studies have documented PCV-3 infection in various organs, including lymphatic organs, heart, and kidney, the detection of the virus in bone suggests a potentially broader tissue tropism than previously recognized. At this stage, it remains unclear whether the PCV-3-associated lesions in bone result from direct viral infection of bone-resident cells (e.g., osteoblasts, osteoclasts, or osteocytes) or whether they are mediated by infected macrophages, which are ubiquitously present across various tissues and may contribute to the multisystemic distribution of lesions. Similarly, vascular involvement in the tunica media and tunica adventitia may be due either to direct viral effects on smooth muscle cells or to secondary immune-mediated mechanisms.

Further investigations employing double-labeling techniques or immunohistochemical detection of viral proteins at the cellular level, which have not yet been successful, are needed to clarify the cellular tropism of PCV-3. It is also worth noting that other researchers have previously reported PCV-3-associated lesions involving skeletal structures, including chondrocytes in the lung,^9^ as well as skeletal muscle with inflammatory lesions. However, these findings have not yet been further investigated or fully described in the context of PCV-3 infection.^33,34,38^ Consequently, pathomorphological investigations, including the detection of PCV-3 in skeletal tissues, are recommended for pigs exhibiting bone lesions, such as chronic rib fractures and “humpy-back” posture.

Since the first reports of PCV-3-associated disease, its presence in lesions has been infrequently documented, with most studies relying on PCR for detection. However, PCR alone is not sufficient for diagnosing the disease. This study, which utilized histological analysis, qPCR, and ISH, provides new evidence suggesting that PCV-3 may be associated with musculoskeletal lesions. This highlights the importance of combining different diagnostic approaches to confirm the pathogenetic role of the virus. Other researchers^5^ have also reported facial edema in cases of PCV-3, a finding that aligns with our observations suggesting that this could be an additional clinical manifestation associated with PCV-3 infection.

In addition to histological lesions compatible with PCV-3-SD, we detected a high amount of PCV-3 DNA by qPCR in multiple organs of finisher pigs, including the CNS. While other studies have detected viral DNA in finishers and pigs ready for slaughter,^28,43,47,57,58^ both with and without clinical signs, this study is the first to document PCV-3-associated lesions histologically and to identify viral mRNA within affected tissue through ISH in this age category.

In contrast to a recently published Swiss study on retrospective assessment of porcine abortions, in which only ISH was performed,^18^ in this study, we detected PCV-3 by qPCR in one-third of the porcine abortion cases. This finding suggests a potential role of PCV-3 in reproductive failure in Switzerland, which has already been described in other countries.^3,5,35,48,49^ The results confirm a previous study^45^ that observed a higher abundance of PCV-3 DNA in fetuses from the final third of gestation compared to those from the second third, with a broader tissue distribution. However, it is important to note that the case load in this study was limited to aborted fetuses from the final third of gestation and sourced exclusively from a single farm (farm A), which restricts the ability to directly compare with earlier stages of pregnancy.

In cases with anamnestic CNS signs, other potentially related infectious agents were not investigated. Among these, atypical porcine pestivirus could be considered within the differential diagnosis, at least for congenital tremors in piglets. However, the pathological changes typically associated with atypical porcine pestivirus (hypomyelination) do not correspond to the findings in this study. Specifically, piglets with atypical porcine pestivirus-associated congenital tremors do not display the histological lesions observed here, particularly the (peri-)arteritis and associated vascular changes.^39,50^ Furthermore, one animal exhibiting clinical CNS signs; macroscopic and histological lesions consistent with PCV-3-SD, including rib fractures with callus formation; and with a high viral load by qPCR underwent next-generation sequencing (data not shown) of a rib sample. The sequencing revealed the presence of PCV-3 strain 29160 (complete genome) as the sole identified pathogen.

The rib fractures observed in the piglets appeared to be consistent with hypoxic damage, potentially leading to fractures.^51^ We speculate that the “humpy-back” posture may be more related to pain induced by the rib fracture, as the pigs seemed to adopt a lordotic position in the thoracic region to alleviate pressure on the chest.^24^ Notably, in contrast to the ribs, the spine and individual vertebrae of these animals did not show any macroscopic or histological lesions. We hypothesize that the fractures were at least facilitated by hypoxia, given the marked inflammatory infiltration observed in arteries within the periosteum and surrounding skeletal muscle. Furthermore, there was no anamnestic evidence of traumatic events that could explain the rib fractures in the piglets analyzed, supporting the idea that hypoxic injury could be the underlying cause.

As differential diagnoses, metabolic bone diseases such as rickets and osteodystrophy were considered. However, both macroscopic and histological evaluations did not support these conditions. Histological examination included bone sections both with and without macroscopically visible callus formation. Growth plate morphology was preserved, and mineralization appeared normal, with no histological signs of metabolic bone disease.

Rib fractures were detected only during the post-mortem examination; however, the submitter reported clinical signs of swollen ribs during life, indicating that fractures likely occurred ante-mortem. Whether these fractures arose spontaneously under normal handling conditions or involved other factors cannot be definitively determined based on current data. Notably, fractures were observed exclusively in ribs, without involvement of other bones. Bone density was not assessed radiologically or morphometrically, and biochemical parameters relevant to bone metabolism, such as serum calcium, phosphorus, and vitamin D levels, were not available. This lack of complementary diagnostic data limits the ability to fully elucidate the pathogenesis of these fractures. As such, our interpretation remains focused on the morphological findings, while acknowledging that metabolic alterations may have acted as predisposing or exacerbating factors. Therefore, the precise mechanisms underlying rib fractures in the context of PCV-3 infection remain to be clarified. Further research is needed to investigate whether the fractures result directly from viral effects on bone or secondary consequences such as hypoxia or immune-mediated vascular damage.

In contrast, older finisher pigs did not display rib fractures (or potentially healed fractures) but instead exhibited lesions in the vertebral bodies, characterized by irregular structure, absence of the intervertebral disk, direct contact between adjacent epiphyseal cartilages, and mild narrowing of the spinal canal due to dorsal cartilaginous proliferation. We speculate that these vertebral lesions may have arisen from improper mechanical burden, probably due to rib fractures sustained earlier in life. However, histological analysis revealed no PCV-3-related lesions in the affected bones of these pigs. Instead, lesions consistent with PCV-3-SD were observed in various organs. The pathogenesis of the vertebral lesions and rib fractures in relation to the vascular lesions associated with PCV-3 remains speculative and warrants further investigation.

It should be noted that body condition was not systematically documented, particularly for animals sampled by field veterinarians. Therefore, the influence of nutritional status on the macroscopic perception of kyphosis cannot be entirely ruled out and may represent a potential source of sampling bias.

Finally, the spatial overlap between PCV-3-associated lesions and skeletal abnormalities raises the possibility of an interaction. However, based on current evidence, this co-occurrence should be interpreted with caution. A direct causal relationship remains speculative, as similar skeletal changes could also arise from non-infectious causes such as mechanical stress. Further studies are needed to clarify whether a pathophysiological link exists.

The results of this study contribute to the advancement of current knowledge on PCV-3 infection and expand the data on lesions potentially associated with this viral infection. (Peri-)arteritis has been well-documented in the literature as a characteristic histopathological finding in PCV-3-SD. The present study supports these previous findings, providing additional evidence linking PCV-3 to these specific systemic vascular lesions.

## Conclusions

This study aimed to investigate the occurrence of PCV-3 infection and its association with clinical signs and lesions in pigs exhibiting spinal deformities, neurological signs, and a “humpy-back” posture. Using a combination of qPCR, ISH, and histological analysis, we assessed tissues from 36 pigs across various age categories. This approach provides new insights into the prevalence, tissue distribution, and pathogenic potential of PCV-3 in pigs with musculoskeletal involvement.

The histological lesions matched the descriptions of PCV-3-SD. Notably, this study is the first to report the detection of PCV-3 DNA in osseous tissues, expanding the spectrum of lesions potentially associated with the virus. Although a direct causal link between PCV-3 and skeletal deformities such as “humpy-back” posture remains unproven, the spatial association with vascular and periosteal lesions suggests a possible contributory role.

Given the overlap between viral detection and inflammatory vascular changes, as well as the presence of chronic bone lesions, pathomorphological investigations including molecular testing for PCV-3 are recommended in pigs with comparable clinical presentations. Further studies are necessary to determine the cellular targets and pathophysiological mechanisms of PCV-3, and to clarify whether the virus plays a direct or indirect role in musculoskeletal pathology.

## Supplemental Material

sj-pdf-1-vet-10.1177_03009858251386914 – Supplemental material for Concurrent histological lesions and molecular detection of porcine circovirus 3 in pigs with skeletal abnormalities and humpy-back posture Supplemental material, sj-pdf-1-vet-10.1177_03009858251386914 for Concurrent histological lesions and molecular detection of porcine circovirus 3 in pigs with skeletal abnormalities and humpy-back posture by Giuliana Rosato, Grace M. Makoni, Àlex Cobos, Marina Sibila, Joaquim Segalés, Robert Graage, Dolf Kümmerlen, Thomas Echtermann, Nadja Aeberhard, Hanna Marti, Barbara Helminger and Frauke Seehusen in Veterinary Pathology
