# Supplementary material for: Concurrent histological lesions and molecular detection of porcine circovirus 3 in pigs with skeletal abnormalities and humpy-back posture
Source: Vet Pathol. 2025 Nov 6;63(2):255–64. doi: 10.1177/03009858251386914 (PMC12882977; doi:10.1177/03009858251386914)
Supplement: sj-pdf-1-vet-10.1177_03009858251386914 – Supplemental material for Concurrent histological lesions and molecular detection of porcine circovirus 3 in pigs with skeletal abnormalities and humpy-back posture [file sj-pdf-1-vet-10.1177_03009858251386914.pdf]

Supplemental Materials

Concurrent histological lesions and molecular detection of porcine circovirus 3 in pigs with skeletal abnormalities and humpy-back posture

Giuliana Rosato, Grace Makanaka Makoni, Àlex Cobos, Marina Sibila, Joaquim Segalés, Robert Graage, Dolf Kümmerlen, Thomas Echtermann, Nadja Aeberhard, Hanna Marti, Barbara Helminger and Frauke Seehusen*

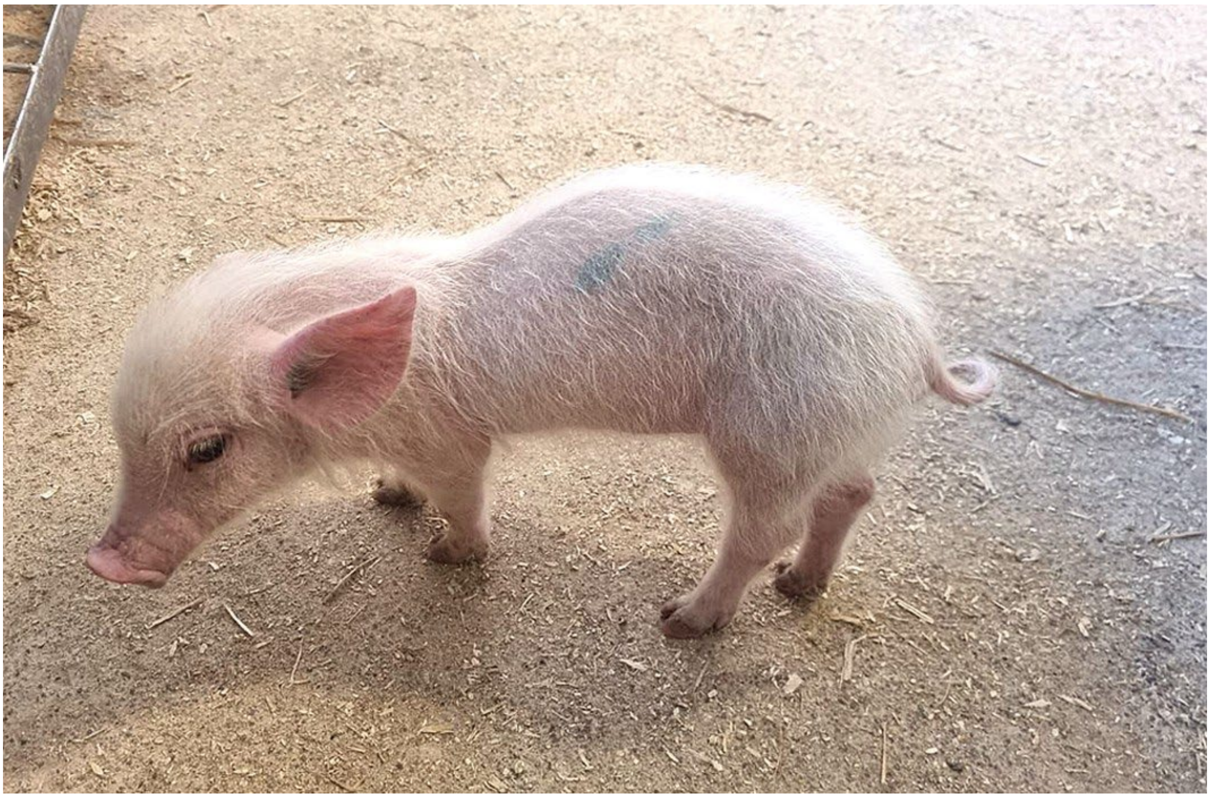

Supplemental Figure S1. Clinical presentation of a 2-week-old suckling piglet exhibiting characteristic postural abnormalities. The animal displays a pronounced "humpy back” posture and caudally rotated ears ("Dumbo ears").

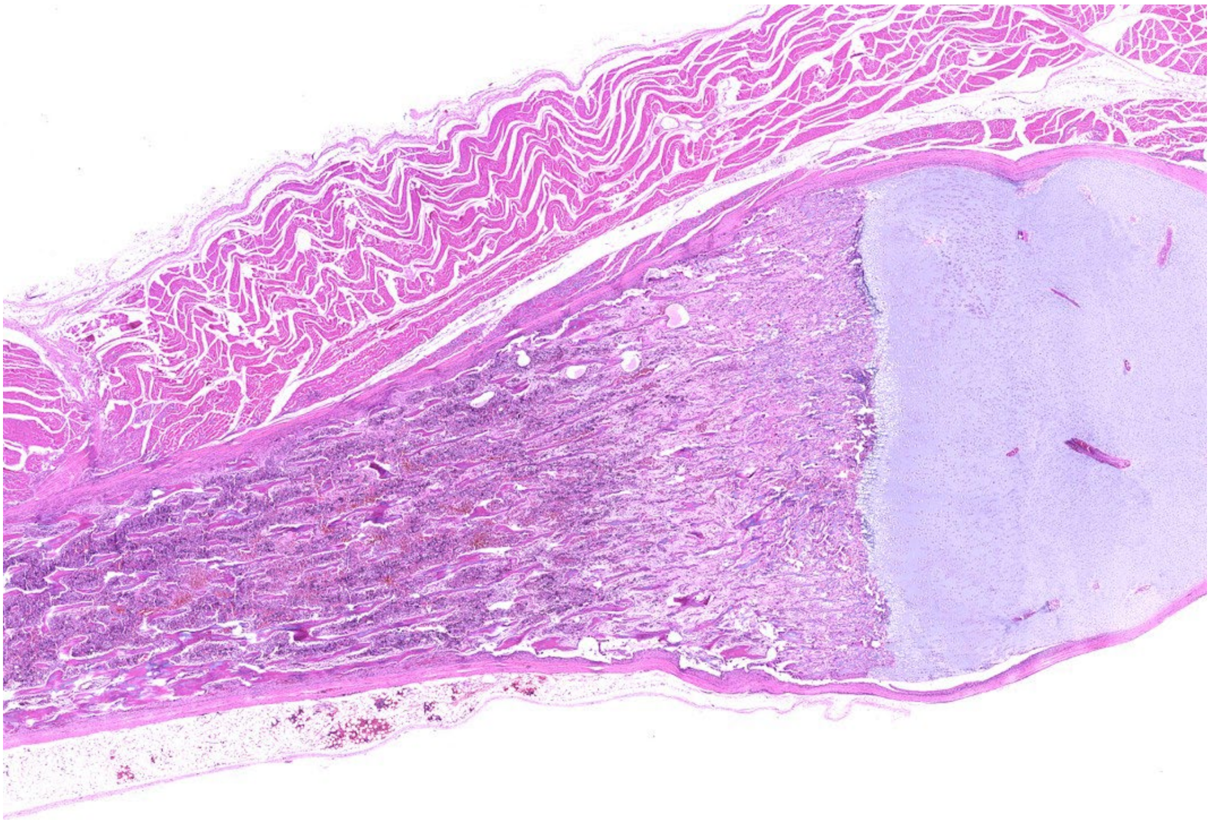

Supplemental Figure S2. Histological section of a non-fractured rib from a 6-week-old piglet that tested quantitative PCR-positive, without macroscopically visible fracture or callus formation. The costochondral junction exhibits intact growth plate architecture. Bone tissue displays normal, physiological mineralization appropriate for the animal's age. No histopathological alterations are evident. Hematoxylin and eosin.

Supplemental Table S1. Data and results for each case, in total 36 cases.

Case	Organ	Age	Farm	Clinical signs	Macroscopic lesions	Histological lesions	PCV-3 viral load (copies/100 ng DNA)	ISH
1	LN	5 W	A	"Humpy-back" posture, edema of the head, "Dumbo ears"	Rib fractures	(Peri-)arteritis compatible with PCV-3-SD; CNS: gliosis and perivascular cuffing; Bo: irregular callus formation, formation of woven bone and chondroid tissue, severe remodeling and myelofibrosis; (peri-)arteritis in surrounding adipose tissue and skeletal muscle	21743	++
	K						20641	++
	H						17425	+
	Bo						134	+
	CNS						146081	N/T
2	LN	4 W	A	"Humpy-back" posture, edema of the head, "Dumbo ears"	Rib fractures	(Peri-)arteritis compatible with PCV-3-SD; CNS: gliosis and perivascular cuffing; Bo: irregular callus formation, formation of woven bone and chondroid tissue, severe remodeling and myelofibrosis; (peri-)arteritis in surrounding adipose tissue and skeletal muscle	149556464	++
	K						244233	++
	Bo						18682	+
	CNS						102496	++
3	LN	5 W	A	Humpy-back posture, edema of the head, "Dumbo ears"	Rib fractures	(Peri-)arteritis compatible with PCV-3-SD; CNS: gliosis and perivascular cuffing; Bo: irregular callus formation, formation of woven bone and chondroid tissue, severe remodeling and myelofibrosis; (peri-)arteritis in surrounding adipose tissue and skeletal muscle	54984	++
	K						33094	+
	Bo						101	N/T
	CNS						60945	++
4	LN	Abortion	A	Late term abortion	None	None	10	N/T
	K						15	N/T
	H						0	N/T
5	Ln	Abortion	A	Late term abortion	None	None	33	N/T
	K						9	N/T
	H						0	neg
6	LN	Abortion	A	Late term abortion	None	None	0	neg
	K						0	neg
	H						0	neg
7	LN	Abortion	A	Late term abortion	None	None	0	N/T
	K						0	N/T
	H						0	N/T

Case	Organ	Age	Farm	Clinical signs	Macroscopic lesions	Histological lesions	PCV-3 viral load (copies/100 ng DNA)	ISH
8	LN	Abortion	A	Late term abortion	None	None	0	N/T
	K						0	N/T
	H						0	N/T
9	LN	Abortion	A	Late term abortion	None	None	0	N/T
	K						0	N/T
	H						0	N/T
10	LN	Abortion	A	Late term abortion	None	None	0	N/T
	K						0	N/T
	H						0	N/T
11	LN	4 d	Other	Swollen head, Dumbo ears	None	(Peri-)arteritis compatible with PCV-3-SD	20461	++
	K						15486	+
	H						60266	++
12	LN	4D	Other	Swollen head, Dumbo ears	None	(Peri-)arteritis compatible with PCV-3-SD	1545644	++
	K						216120	++
	H						4551509	++
13	LN	Finisher	Other	Abnormal spinal curvature	Absence intervertebral disc L1-L2; L1 irregular vertebral body with widened ventral contour; L2 ventrally shortened, wedge-shaped vertebra. Narrowing of spinal canal by cartilaginous proliferations	(Peri-)arteritis in kidney; Bo: (Peri-)arteritis in surrounding adipose tissue and skeletal muscle	415	N/T
	K						68	neg
	H						45	N/T
	Bo						0	N/T
14	LN	Ready for slaughter	Farm A, slaughtered in house	Mild abnormal spinal curvature	None	(Peri-)arteritis compatible with PCV-3-SD; CNS: perivascular cuffing	1986	N/T
	K						588	neg
	H						596	N/T
	CNS						7452	neg
	Bo						0	N/T

Case	Organ	Age	Farm	Clinical signs	Macroscopic lesions	Histological lesions	PCV-3 viral load (copies/100 ng DNA)	ISH
15	LN	Ready for slaughter	Farm A, slaughtered in house	None	None	(Peri-)arteritis compatible with PCV-3-SD; CNS: perivascular cuffing	6499	+
	K						2716	+
	H						79	N/T
	CNS						183576	N/T
16	LN	Ready for slaughter	Farm A, slaughtered in house	None	None	(Peri-)arteritis compatible with PCV-3-SD	44	N/T
	K						38	N/T
	H						46	N/T
17	LN	Ready for slaughter	Farm A, slaughtered in house	None	None	(Peri-)arteritis compatible with PCV-3-SD	7	N/T
	K						26	N/T
	H						19	N/T
18	LN	runt-finisher	Other	"Humpy-back" posture	None	(Peri-)arteritis compatible with PCV-3-SD; CNS: perivascular cuffing	20893	N/T
	K						4217	N/T
	H						93247	N/T
19	LN	5 W	A	Gait abnormalities	None	CNS: perivascular cuffing, glia nodules	0	N/T
	K						0	N/T
	H						0	N/T
	CNS						0	N/T
20	LN	7 W	A	Gait abnormalities primarily affecting the hind limbs, CNS symptoms	Rib fractures	(Peri-)arteritis compatible with PCV-3-SD; CNS: meningoencephalitis, gliosis and perivascular cuffing; Bo: irregular callus formation, formation of woven bone and chondroid tissue, severe remodeling and myelofibrosis; (peri-)arteritis in surrounding adipose tissue and skeletal muscle	56843	+
	K						5580	N/T
	H						944	N/T
	CNS						2319	neg
	Bo						791155	N/T
21	LN	6 W	A	Gait abnormalities primarily affecting the hind limbs, CNS symptoms	Rib fractures	(Peri-)arteritis compatible with PCV-3-SD; CNS: meningoencephalitis, gliosis and perivascular cuffing; Bo: irregular callus formation, formation of woven bone and chondroid tissue, severe remodeling and myelofibrosis; (peri-)arteritis in surrounding adipose tissue and skeletal muscle	8	neg
	K						0	N/T
	H						4	N/T
	CNS						0	neg
	Bo						127	N/T

Case	Organ	Age	Farm	Clinical signs	Macroscopic lesions	Histological lesions	PCV-3 viral load (copies/100 ng DNA)	ISH
22	LN	4 W	Other	Severe waisting, "Humpy-back" posture, "Dumbo ears"	Rib fractures	(Peri-)arteritis compatible with PCV-3-SD; CNS: gliosis and perivascular cuffing; Bo: irregular callus formation, formation of woven bone and chondroid tissue, severe remodeling and myelofibrosis; (peri-)arteritis in surrounding adipose tissue and skeletal muscle	6142	N/T
	K						13915	N/T
	H						87603	N/T
	CNS						3753	++
	Bo						9059	N/T
23	LN	9 W	Other	Severe waisting, "Humpy-back" posture, "Dumbo ears"	Rib fractures	(Peri-)arteritis compatible with PCV-3-SD; CNS: gliosis and perivascular cuffing; Bo: irregular callus formation, formation of woven bone and chondroid tissue, severe remodeling and myelofibrosis; (peri-)arteritis in surrounding adipose tissue and skeletal muscle	1322	N/T
	K						2395	N/T
	H						12678	N/T
	CNS						7562	N/T
	Bo						14	N/T
24	LN,K,H	2 d	Other	Congenital tremor, "Humpy-back" posture, "Dumbo ears", edema of the head	None	(Peri-)arteritis compatible with PCV-3-SD	11142096	++
25	LN,K,H	5-6 W	A	Kyphosis	None	(Peri-)arteritis compatible with PCV-3-SD	8886	neg
26	LN,K,H	5-6 W	A	Kyphosis	None	(Peri-)arteritis compatible with PCV-3-SD	20229	++
27	CNS	6 W	A	"Humpy-back" posture, abnormal spinal curvature	Rib fractures	(Peri-)arteritis compatible with PCV-3-SD; CNS: perivascular cuffing; Bo: irregular callus formation, formation of woven bone and chondroid tissue, severe remodeling and myelofibrosis; (peri-)arteritis in surrounding adipose tissue and skeletal muscle	49081	N/T
	LN						35	+
	K						586	neg
	H						276	neg
	Bo						14	+
28	CNS	6 W	A	"Humpy-back" posture, abnormal spinal curvature	Rib fractures	(Peri-)arteritis compatible with PCV-3-SD; CNS: perivascular cuffing; Bo: irregular callus formation, formation of woven bone and chondroid tissue, severe remodeling and myelofibrosis; (peri-)arteritis in surrounding adipose tissue and skeletal muscle	1435	++

Case	Organ	Age	Farm	Clinical signs	Macroscopic lesions	Histological lesions	PCV-3 viral load (copies/100 ng DNA)	ISH
29	LN	6 W	A	"Humpy-back" posture, abnormal spinal curvature	Rib fractures	(Peri-)arteritis compatible with PCV-3-SD; Bo: irregular callus formation, formation of woven bone and chondroid tissue, severe remodeling and myelofibrosis; (peri-)arteritis in surrounding adipose tissue and skeletal muscle	14185	N/T
	Bo						0	N/T
30	LN,K,H	1 W	A	Swollen ribs	Rib fractures	(Peri-)arteritis compatible with PCV-3-SD; Bo: irregular callus formation, formation of woven bone and chondroid tissue, severe remodeling and myelofibrosis; (peri-)arteritis in surrounding adipose tissue and skeletal muscle	269907	+
	Bo						271879	N/T
31	H	Abortion	A	Late term abortion	None	None	0	N/T
	LN						16943	N/T
	K						130	N/T
32	LN,K,H	5 d	A	None	None	None	0	N/T
33	LN,K,H	2 W	Other	CNS symptoms	None	None	0	N/T
34	LN,K,H	2 W	Other	CNS symptoms	None	None	0	N/T
35	K	1 d	Other	No macroscopic lesions	None	None	0	N/T
	H						0	N/T
	LN						0	N/T
36	K	Abortion	A	Late term abortion	None	None	0	N/T
	LN, H						0	N/T

Abbreviations: PCV-3, porcine circovirus 3; ISH, *in situ* hybridization; LN, lymph node; K, kidney; H, heart; Bo, bone; CNS, brain or spinal cord; d, day, W, week; neg, absent PCV-3 ISH signal; +, moderate PCV-3 ISH-positive signal; ++, marked PCV-3 ISH-positive signal; PCV-3-SD, PCV-3-systemic disease; N/T, not tested.
